# Supplementary material for: Characterization of Recurrent Relevant Genes Reveals a Novel Role of RPL36A in Radioresistant Oral Squamous Cell Carcinoma
Source: Cancers (Basel). 2021 Nov 10;13(22):5623. doi: 10.3390/cancers13225623 (PMC8616119; doi:10.3390/cancers13225623)
Supplement: Supplementary file 1 [file cancers-13-05623-s001.zip › Supplementary Table 1 and 2/Supplementary Table 2.pdf]

Supplementary Table S2. Results of univariate and multivariate analyses of disease-specific survival of 136 OSCC patients with radiotherapy from the validation-testing Taiwanese cohort

	Univariate			Multivariate		
Variables	Hazard ratio	95% CI	*p* value	Hazard ratio	95% CI	*p* value
Age, years[a]	0.972	0.945-1.000	0.045[b]	0.973	0.945-1.003	0.073
Sex						
Male	Reference			Reference		
Female	1.444	0.521-4.003	0.502	0.710	0.131-3.839	0.691
Smoking						
No	Reference			Reference		
Yes	0.628	0.226-1.741	0.402	0.395	0.068-2.289	0.300
Overall TNM stage						
I-II	Reference			Reference		
III-IV	0.785	0.383-1.607	0.519	0.550	0.246-1.229	0.145
Node classification						
n≤1	Reference			Reference		
n>1	1.843	1.084-3.133	0.025[b]	2.262	1.239-4.130	0.008[b]
RPL36A						
Low	Reference			Reference		
High	1.721	0.998-2.969	0.048[b]	1.782	1.021-3.111	0.042[b]

Abbreviations: CI, confidence interval; TNM, tumor, node, metastasis.

[a]Continuous variable, [b]Statistically significant.
