# Supplementary figures and images for: Characterization of Recurrent Relevant Genes Reveals a Novel Role of RPL36A in Radioresistant Oral Squamous Cell Carcinoma
Source: Cancers (Basel). 2021 Nov 10;13(22):5623. doi: 10.3390/cancers13225623 (PMC8616119; doi:10.3390/cancers13225623)

Fig. 4C

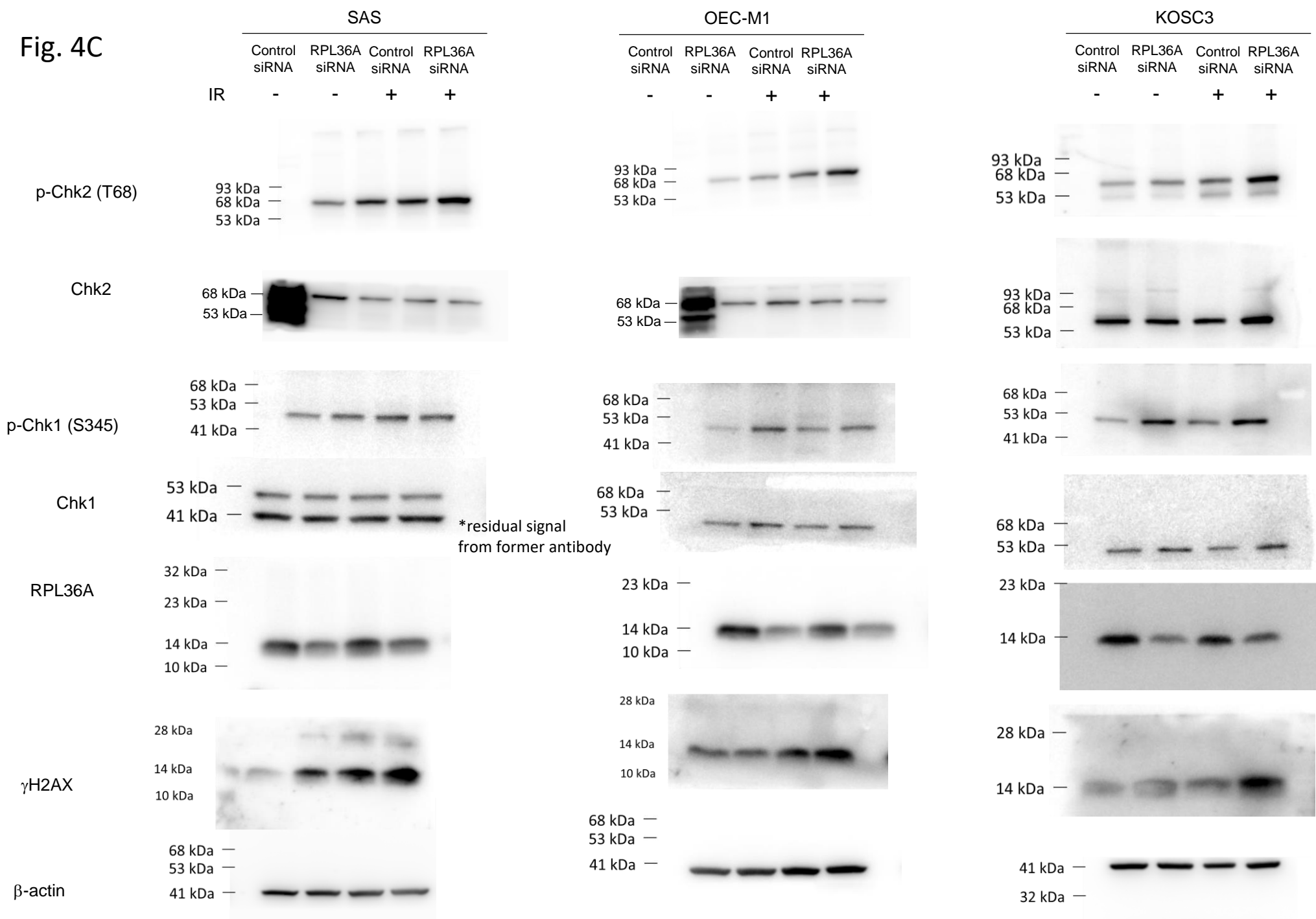

Fig. 5C

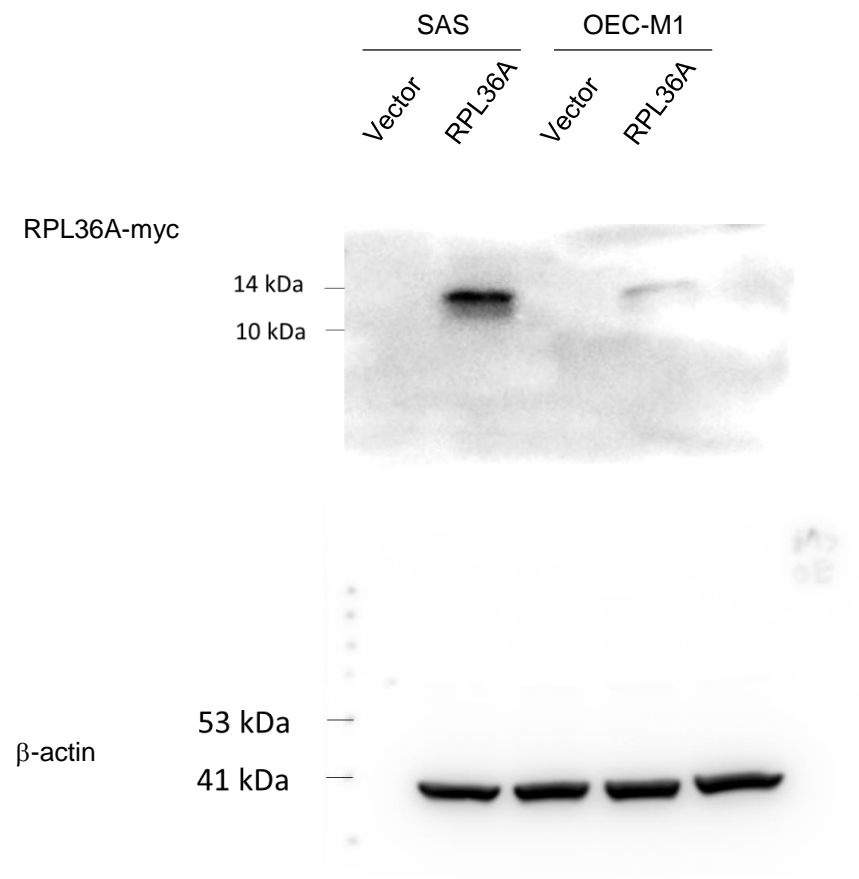

Supplement: Supplementary file 1 [file cancers-13-05623-s001.zip › Supplementary Table 1 and 2/Supplementary Figure 1.pdf]
